# Comparative Proteomic Analysis of Mature Pollen in Triploid and Diploid *Populus deltoides*

**DOI:** 10.3390/ijms17091475

**Published:** 2016-09-03

**Authors:** Xiao-Ling Zhang, Jin Zhang, Ying-Hua Guo, Pei Sun, Hui-Xia Jia, Wei Fan, Meng-Zhu Lu, Jian-Jun Hu

**Affiliations:** 1 State Key Laboratory of Tree Genetics and Breeding, Key Laboratory of Tree Breeding and Cultivation of State Forestry Administration, Research Institute of Forestry, Chinese Academy of Forestry, Beijing 100091, China; ls_wu@caf.ac.cn (X.-L.Z.); yh_guo@caf.ac.cn (Y.-H.G.); sun_caf@sina.com (P.S.); huixia__jia@126.com (H.-X.J.); atom201001@163.com (W.F.); lumz@caf.ac.cn (M.-Z.L.); 2 Collaborative Innovation Center of Sustainable Forestry in Southern China, Nanjing Forestry University, Nanjing 210037, China

**Keywords:** poplar, pollen, diploid, triploid, allergenic protein, proteomics

## Abstract

Ploidy affects plant growth vigor and cell size, but the relative effects of pollen fertility and allergenicity between triploid and diploid have not been systematically examined. Here we performed comparative analyses of fertility, proteome, and abundances of putative allergenic proteins of pollen in triploid poplar ‘ZhongHuai1’ (‘ZH1’, triploid) and ‘ZhongHuai2’ (‘ZH2’, diploid) generated from the same parents. The mature pollen was sterile in triploid poplar ‘ZH1’. By applying two-dimensional gel electrophoresis (2-DE), a total of 72 differentially expressed protein spots (DEPs) were detected in triploid poplar pollen. Among them, 24 upregulated and 43 downregulated proteins were identified in triploid poplar pollen using matrix-assisted laser desorption/ionisation coupled with time of-flight tandem mass spectrometer analysis (MALDI-TOF/TOF MS/MS). The main functions of these DEPs were related with “S-adenosylmethionine metabolism”, “actin cytoskeleton organization”, or “translational elongation”. The infertility of triploid poplar pollen might be related to its abnormal cytoskeletal system. In addition, the abundances of previously identified 28 putative allergenic proteins were compared among three poplar varieties (‘ZH1’, ‘ZH2’, and ‘2KEN8‘). Most putative allergenic proteins were downregulated in triploid poplar pollen. This work provides an insight into understanding the protein regulation mechanism of pollen infertility and low allergenicity in triploid poplar, and gives a clue to improving poplar polyploidy breeding and decreasing the pollen allergenicity.

## 1. Introduction

Polyploidy is having more than two sets of chromosomes present within a nucleus, which is a widespread phenomenon in the plant kingdom. Polyploidization frequently occurs in plants, and it has been regarded as one of the major speciation mechanisms [1]. Polyploidy has considerable effects on the expression of genes and abundance of proteins [2]. Compared with diploid, polyploidy shows advantages in many aspects, such as increasing in biomass and fruit yield [3]. Polyploidy also provides genome buffering, as it is helpful to increase the allelic diversity and then generate novel phenotypic variation [4]. However, most of the polyploidy appears to decrease pollen fertility and reduced seed amount. Meirmans et al. found that triploid dandelion did not produce more seeds or heavier seeds, and it was due to male sterility because of disorder of its nuclear genes [5].

As an important biomass and feedstock plant species, poplar provides a wide range of industrial construction woods [6]. Since Nilsson-Ehle found the giant natural European triploid aspen (*Populus tremula*) [7], more and more natural or artificial polyploidy poplar species were reported and applied into breeding. Cai et al. produced tetraploid *P. pseudo*-*simonii* from leaf explants of diploid *P. pseudo*-*simonii* by colchicine treatment. The size and density of leaf stomata in tetraploid poplar were significantly greater than that of diploid [8]. At present, the study of the low fertility of polyploidy has mainly focused on the morphological observation. The polyploid medicinal plant *Pinellia ternata* (Araceae) has low fertility and reduced amount of seeds because of abnormal meiotic division [9], which is similar to that of *Arabidopsis*, rice, and wheat [10,11,12]. However, the molecular characteristics of pollen in polyploid poplar have not been revealed. 

Between April and May, male blossoming of Salicaceae results in amounts of pollen spread into the air, which seriously affects human health [13]. As the main allergens in pollen, the allergenic proteins will hydration quickly and to elicit the allergic reaction in a short time. Immune electron microscopy of dry and rehydrated birch pollen showed that Bet v I, a major allergen in birch pollen, can migrate into the exine and to the surface of pollen grains after brief hydration [14]. At present, the study on the pollen allergy is mainly diagnosed through testing the allergic reaction of the patients’ serums. Several studies have indicated that the main allergies varied in different cities. In New York City, NY, U.S., 371 allergic patients’ serums were tested and the highest rates of allergies were derived from *Quercus* spp. (34.3%), *Betula* spp. (32.9%), and *Acer* spp. (32.9%), followed by *Populus* spp. (20.6%) and *Ulmus* spp. (24.6%) [15]. Erkara surveyed the pollen grains in the atmosphere of Sivrihisar, Turkey for two continuous years, and the result showed that the majority of the allergenic pollen grains were from *Pinaceae*, *Cupressaceae*, *Fraxinus* spp., *Cedrus* spp., *Populus* spp., and so on [16]. In Talca, Chile, the highest number of airborne pollen grains in the atmosphere was from *Platanus acerifolia* (203 grains/m^3^, day), and *Populus* spp. had a maximum weekly daily average 103 grains/m^3^ [17].

Proteomics is a powerful tool to understand the dynamic changes of proteins [18]. In this study, we compared the differences of pollen germination and proteome between two *P. deltoides* varieties, ‘ZhongHuai1’ (‘ZH1’, triploid) and ‘ZhongHuai2’ (‘ZH2’, diploid), which were generated from the same parents (*P. deltoides* ‘55/56’ × *P. deltoides* ‘Imperial’) [19,20,21]. In our previous study, a total of 28 putative allergenic proteins were identified from mature pollen of diploid *P. deltoides* CL. ‘2KEN8’ using proteome research approach [22]. Here, the protein abundances and gene expression levels of the 28 putative allergenic proteins were compared among the three varieties (‘ZH1’, ‘ZH2’, and ‘2KEN8’). This study is helpful for understanding the molecular mechanism of differences in pollen fertility and allergenicity between triploid and diploid poplar.

## 2. Results

**Figure 1:**
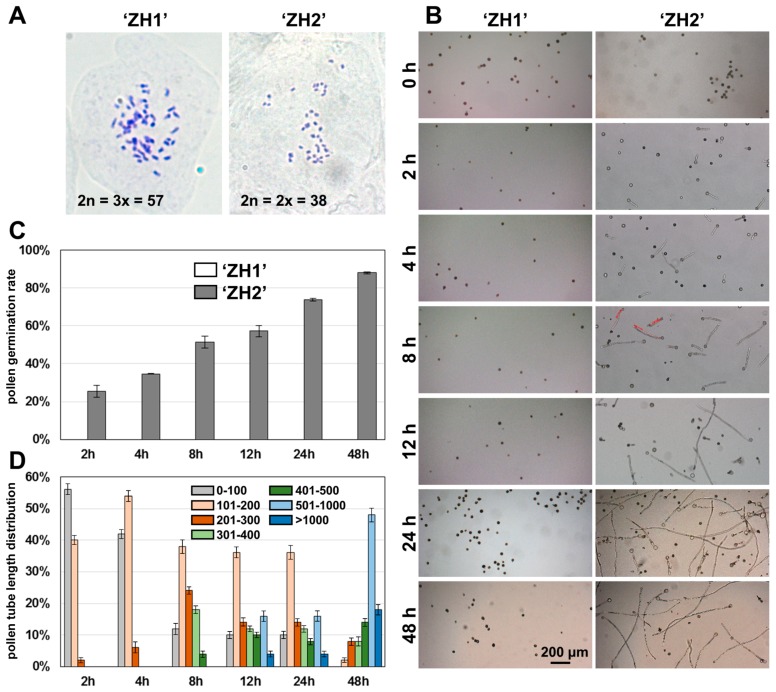
Pollen germination of triploid ‘ZH1’ and diploid ‘ZH2’ *P. deltoides*. (**A**) Somatic chromosome number in triploid poplar ‘ZH1’ and diploid poplar ‘ZH2’; (**B**) pollen tube growth of ‘ZH1’ and ‘ZH2’ during the germination (2, 4, 8, 12, and 24 h); (**C**) pollen germination rate of ‘ZH1’ and ‘ZH2’; (**D**) pollen tube length distribution of ‘ZH2’ pollen during the germination.

In our previous study, we identified a triploid *P. deltoides* variety ‘ZH1’ and a diploid variety ‘ZH2’ from the same parents (*P. deltoides* ‘55/56’ × *P. deltoides* ‘Imperial’), and the ploidy levels of the hybrids were determined using flow cytometric analysis [21]. To confirm the ploidy level of the two varieties, we further analyzed the karyotypes of the two materials. As shown in Figure 1A, the chromosome numbers of ‘ZH1’ and ‘ZH2’ were 2*n* = 3*x* = 57 and 2*n* = 2*x* = 38, respectively. The result showed that ‘ZH1’ was triploid and ‘ZH2’ was diploid. 

### 2.1. Pollen Germination in Triploid and Diploid P. deltoides

To compare the pollen fertility of triploid and diploid poplar, the pollen of ‘ZH1’ and ‘ZH2’ was germinated in vitro (Figure 1B). After 2 h cultivation, the pollen of ‘ZH2’ began to germinate, and the germination rate was increased as a function of culture time. Over 88% pollen of diploid ‘ZH2’ was germinated when the pollen was cultivated for 48 h, while the pollen of triploid ‘ZH1’ was not germinated at all, even after 48 h cultivation (Figure 1B,C). The length of pollen tubes in different culture times of diploid ‘ZH2’ was measured, which increased with the elongation of culture time. At the beginning 4 h, most pollen tubes of ‘ZH2’ were distributed, ranging from 0 to 200 μm. After 48 h cultivation, most pollen tubes of ‘ZH2’ had a length of 500–1000 μm (Figure 1D).

### 2.2. Two-Dimensional Gel Electrophoresis (2-DE) Analysis and Identification of Differentially Expressed Proteins

**Figure 2:**
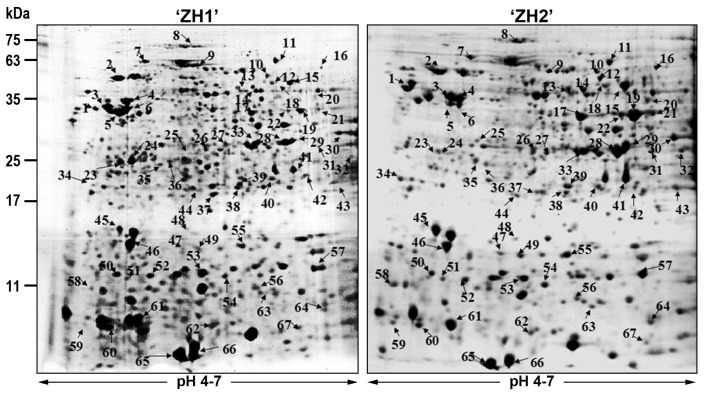
Two-dimensional gel electrophoresis (2-DE) image analysis of triploid ‘ZH1’ and diploid ‘ZH2’ *P. deltoides*. The numbers with arrows indicate the differentially expressed protein spots (DEPs) with *p* < 0.05.

As shown in Figure 2, the pollen proteins of ‘ZH1’ and ‘ZH2’ were separated using 2-DE. A total of 557 and 598 repeatable protein spots were identified from ‘ZH1’ and ‘ZH2’ mature pollen, respectively. Among these protein spots, 488 were matched in the two varieties’ mature pollen. The matched proteins covered the isoelectric point (pI) ranging from 5.0 to 6.5, and their molecular weight (MW) ranged from 17 to 63 kDa. To compare the differences of mature pollen proteins between triploid ‘ZH1’ and diploid ‘ZH2’, the intensity of each matched spot from three biological replicates was analyzed using ImageMaster 2D Platinum software version 6.0 (GE Healthcare, Little Chalfont, UK), and significantly (*p* ≤ 0.05) altered spots were identified with the Student’s *t*-test. A total of 72 repeatable differentially expressed protein spots (DEPs) were identified; 22 were upregulated and 50 were downregulated in the mature pollen of triploid poplar ‘ZH1’ (Table 1).

### 2.3. Functional Classification of DEPs

Subsequently, 67 of the 72 DEPs were successfully identified using MALDI-TOF/TOF MS/MS (Table 1). For most of the identified proteins, the experimental MW and pI were basically consistent with the theoretical values of the identified proteins. These proteins are involved in various metabolic pathways or processes, such as carbohydrate-metabolism- and energy-metabolism-related proteins (34.3%), amino-acid-metabolism-related proteins (7.5%), protein-metabolism-related proteins (32.8%), defense- or stress-related proteins (14.9%), signal transduction proteins (3%), and allergy-related proteins (3%) (Table 1).

**Figure 3:**
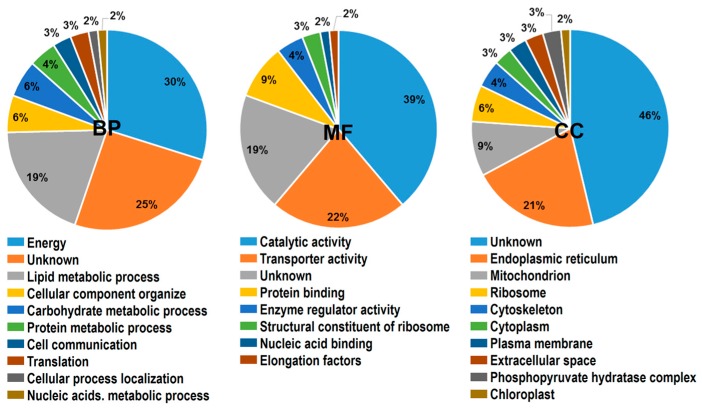
Functional annotation of identified DEPs based on Gene Ontology (GO) categorization. Results are summarized for three main GO categories: biological process (BP), molecular function (MF), and cellular component (CC).

In addition, the 67 proteins were grouped by Gene Ontology (GO) system into biological process (BP), molecular function (MF), and cellular compartment (CC) classes. In terms of BP, 30% of the DEPs were involved in the biological process of energy provision and 19% DEPs were involved in the lipid metabolic process. For MF category, more than half of these proteins belonged to catalytic activity and transporter activity, accounting for 38.80% and 22.39%, respectively. On the basis of predicted subcellular location, the proteins were classified into 10 categories in cellular compartments; about 21% were located in endoplasmic reticulum (Figure 3).

**Figure 4:**
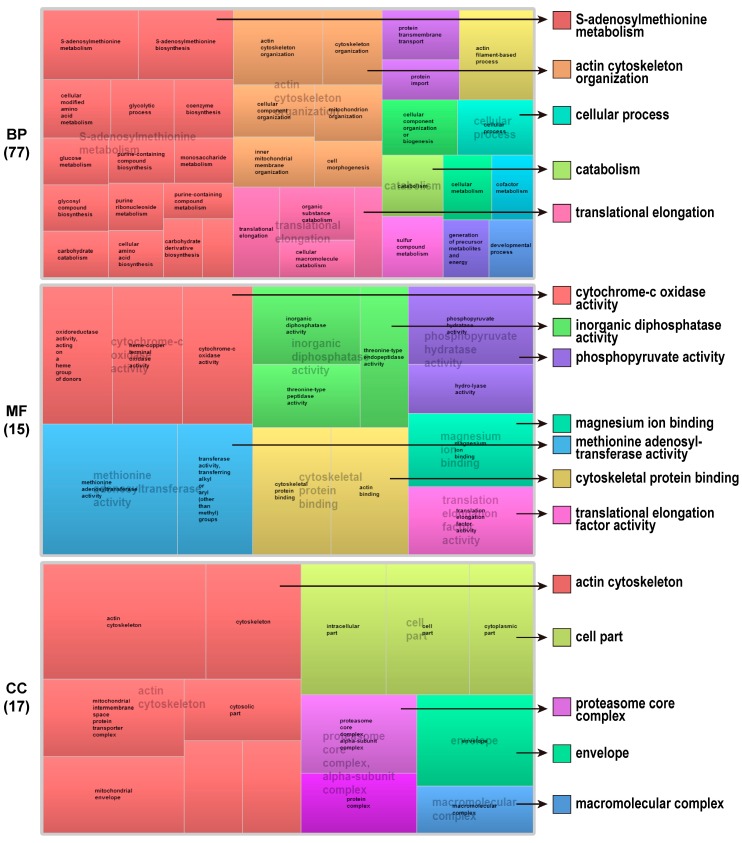
GO treemaps for the DEPs in ‘ZH1’ and ‘ZH2’ pollen. GO terms for proteins identified in ‘ZH1’ and ‘ZH2’ pollen are shown. The box size correlates to the −log10 *P*-value of the GO term. Boxes with the same color can be grouped together and correspond to the same upper-hierarchy GO-term.

In order to further understand functional categories of DEPs, REVIGO reduction analysis tool was used to summarize GO terms together with their *P*-values [23]. According to categories BP, gene clusters represent “S-adenosylmethionine metabolism”, “actin cytoskeleton organization”, “translational elongation”, “cellular processes”, and “catabolism”. Among them, “S-adenosylmethionine metabolism” and “actin cytoskeleton organization” were the main significantly different biological processes between triploid and diploid poplar mature pollen. The MF indicated seven GO terms related with “cytochrome oxidase activity”, “methionine adenosyltransferase activity”, “inorganic diphosphatase activity”, “cytoskeletal protein binding”, “phosphopyruvate hydratase activity”, “magnesium ion binding”, and “translation elongation factor activity”. For categories based on CC, the identified proteins were mainly related with actin cytoskeleton (Figure 4).

### 2.4. Expression Patterns of Identified DEPs

**Figure 5:**
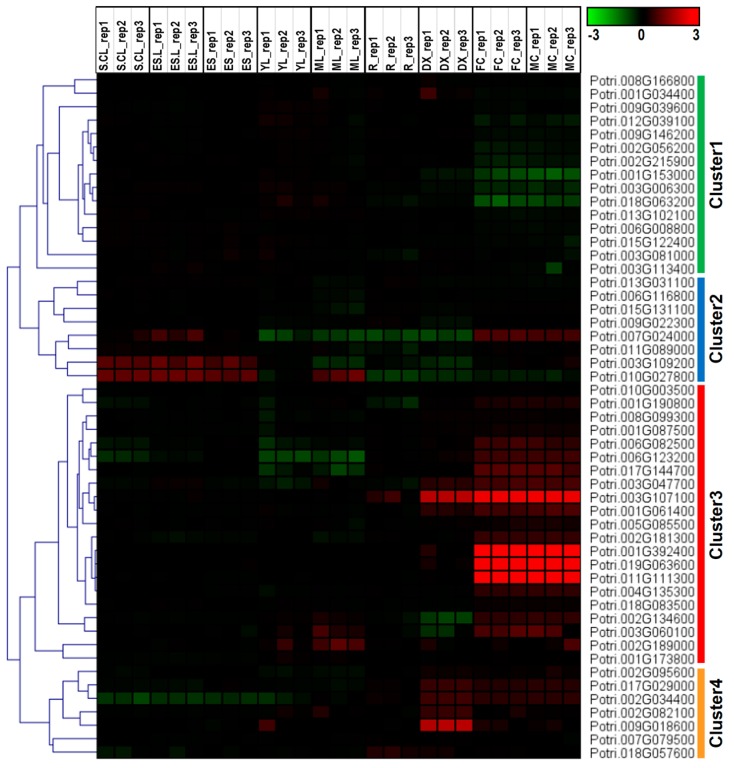
Expression profiles of genes coding DEPs between ‘ZH1’ and ‘ZH2’ mature pollen across different tissues. The Affymetric microarray data were obtained from the National Center for Biotechnology Information (NCBI) Gene Expression Omnibus (GEO) database under the series accession number GSE13990. S.CL—Seedling continuous light; ES.L—Etiolated seedling 6 days transferred to light 3 h; ES—Etiolated seedling; YL—Young leaf; ML—Mature leaf; R—Root; DX—Differentiating xylem; FC—Female catkin; MC—Male catkin. Background-corrected expression intensities were log-transformed and visualized as heatmaps. Color scale represents log2 expression values, green represent low level and red indicates high level of transcript abundance.

To explore the potential roles of the DEPs in poplar developmental processes, we investigated the expression profiles of identified DEPs across various tissues. The majority of these genes showed tissue-specific expression patterns (Figure 5). Based on the expression patterns, the genes were classified into four clusters (Cluster 1–4). As shown in Figure 5, only genes in Cluster 3 were highly expressed in the female and male catkins. Genes in Cluster 1 had relatively low abundance in female and male catkins, genes in Cluster 2 were highly expressed in seedlings (including S.CL—seedling continuous light, ES.L—etiolated seedling 6 days transferred to light 3 h, and ES—etiolated seedling), while genes in Cluster 4 were highly expressed in differentiating xylem.

### 2.5. Comparative Analysis of Predicted Allergenic Proteins

**Figure 6:**
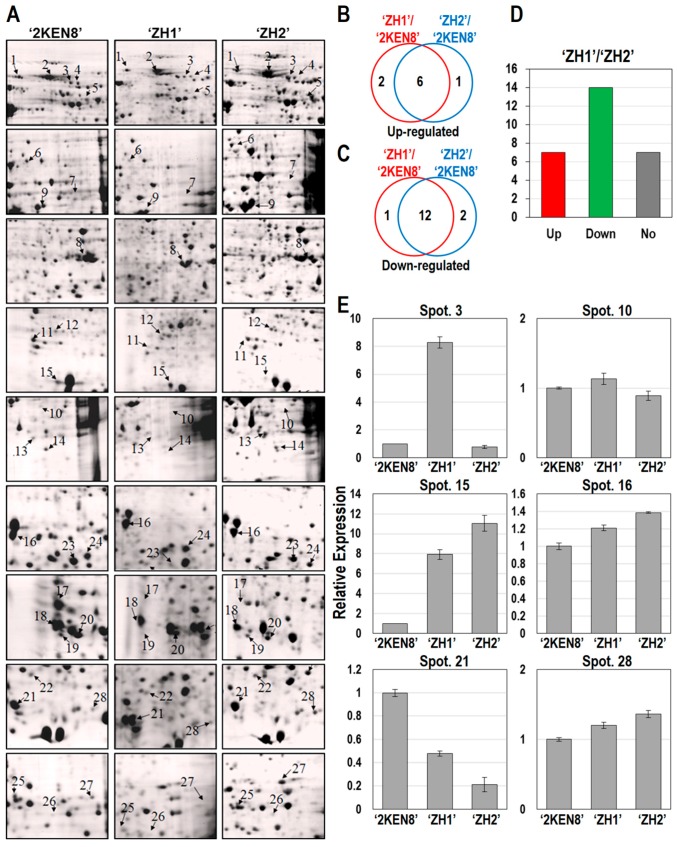
Predicted allergenic proteins in *P. deltoides* ‘ZH1’, ‘ZH2’, and ‘2KEN8’ mature pollen. (**A**) The protein abundance of previously predicted 28 allergenic proteins [22] in three *P. deltoides* cultivars ‘ZH1’, ‘ZH2’, and ‘2KEN8’ mature pollen; Venn diagram of upregulated (**B**) or downregulated (**C**) proteins in ‘ZH1’ and ‘ZH2’ compared with ‘2KEN8’ from (**A**); (**D**) differentially expressed proteins among 28 predicted allergenic proteins between ‘ZH1’ and ‘ZH2’; (**E**) expression analysis of six selected allergen related genes in the three cultivars using qRT-PCR.

In our previous study, we identified 28 candidate allergenic proteins in diploid *P. deltoides* ‘2KEN8’ mature pollen [22]. Here, we compared the abundance of these 28 candidate allergenic proteins among the three varieties (‘ZH1’, ‘ZH2’, and ‘2KEN8’) (Figure 6A and Table 2). The expression profiles of the predicted allergenic proteins in the two varieties (‘ZH1’ and ‘ZH2’) were similar compared with ‘2KEN8’. For example, 6 and 12 predicted allergenic proteins were up- and downregulated in both ‘ZH1’/’2KEN8’ and ‘ZH2’/’2KEN8’, respectively. Overall, most allergenic proteins in ‘ZH1’ and ‘ZH2’ mature pollen have relatively lower abundance than that in ‘2KEN8’ (Figure 6B,C and Table 2). In the triploid ‘ZH1’, 14 predicted allergenic proteins were downregulated and 7 were upregulated when compared with the diploid ‘ZH2’ (Figure 6D). To confirm the expression profiles of the genes coding the putative allergenic proteins, qRT-PCR analysis was performed on ‘ZH1’ and ‘ZH2’ mature pollen for six genes (Figure 6E). Protein expression and gene expression of the six protein spots were analyzed; protein expression and gene expression were almost proportional in relationship.

## 3. Discussion

### 3.1. The Mature Pollen of Triploid Poplar ‘ZH1’ Failed to Germinate

As a widespread biological process, polyploidization has provided much genetic variation for plant adaptive evolution. It not only provides extra gene copies, strengthening the robustness against malignant mutations, but also provides abundant genetic materials for neofunctionalization. Therefore, polyploidy has been considered as an important force in the evolution of plants [24,25,26]. However, polyploidy has always been accompanied by low fertility. Soltis et al. reported that the rates of pollen germination in the 187 triploid grapes ranged from 0% to 5.88%, and about 46% of the 187 triploids showed no germination [21]. Triploid clones of *Hypericum androsaemum* had 0%–6% pollen germination rate, and the seeds of the triploids were fewer than the diploids. In addition, the seeds from the triploids failed to germinate [27]. In this study, we observed that the mature pollen of triploid poplar ‘ZH1’ failed to germinate, which is similar with the findings in many other triploid species. 

The ploidy of plant species will affect the protein abundance in proteomic level. An et al. analyzed the leaf proteomes of cassava diploid and autotetraploid genotypes; 47 upregulated proteins and 5 downregulated proteins were identified in autotetraploid genotype [25]. In our study, the proteomic analysis showed that significant differences in protein expression patterns between triploid and diploid poplar pollen. A total of 72 DEPs were identified, 22 were upregulated and 50 were downregulated in mature pollen of triploid poplar ‘ZH1’. Most of the identified DEPs showed similar molecular weights and pI values between the experimental and the theoretical results (Table 1). The individuals with large differences between experimental and theoretical results (e.g., spot 21) might be caused by the different variants generated by alternative splicing events. Among the DEPs, most proteins were related with “carbohydrate metabolism and energy metabolism”, “amino acid metabolism”, “protein metabolism”, and “defense or stress”. However, in regard to proteins related to the “defense or stress”, there were 9 upregulated and 1 downregulated protein in ‘ZH1’ compared with ‘ZH2’. In addition, most of the DEPs related to defense or stress belong to Cluster 3 and Cluster 4, which were highly expressed in the female and male catkins.

In *Arabidopsis*, ploidy affects various morphological and fitness traits, such as stomata size, flower size, and seed weight [28]. To further understand the function of DEPs that were associated with the sterility of triploid, the annotation and enrichment of the GO was conducted. GO-enrichment analysis revealed that the GO terms related with actin cytoskeleton were significantly enriched in DEPs between triploid and diploid poplar mature pollen (Figure 4). As an important component of pollen grain, cytoskeleton controls not only how the pollen tube grows but also how the cytoplasm dynamically reorganizes during tube elongation [29]. During the fertilization, the pollen tube must be guided to enter the ovule via the micropyle with the involvement of actin filaments and actin-binding proteins. *Arabidopsis* microtubule-associated protein 18 (MAP18) modulates actin filaments to directional cell growth and cortical microtubule organization [30]. Generally, actin filaments in pollen tubes will arrange into higher-order longitudinal actin cables to generate the reverse fountain cytoplasmic streaming pattern. During the process, the actin-depolymerizing factor (ADF7) evolved to promote turnover of longitudinal actin cables by severing actin filaments [31]. The abnormal cytoskeletal systems might be related with low pollen viability and abnormal pollen tube elongation in pollen of triploid poplar ‘ZH1’.

In this study, the ubiquitin proteasome carboxyl terminal hydrolase (spot 24) was upregulated in pollen of triploid poplar ‘ZH1’. In eukaryotic organisms, the ubiquitin carboxyl terminal hydrolytic enzymes are involved in short-life proteins turn over and some abnormal protein degradation pathways, which play important control functions in cell growth, signal transduction, and aspects such as plant senescence [32,33,34]. Lin et al. found that ubiquitin carboxyl terminal hydrolase in sterile lines was highly expressed, and speculated that the ubiquitin–proteasome pathway is closely related to wheat male sterility [35]. Sequence alignment showed that the protein in spot 39 shares 71.1% similarity with a 24 kDa protein of FoF1-ATP complex in *Pyrus bretschneideri* (not shown). FoF1-ATP synthase is the key enzyme in the oxidative phosphorylation and phosphorylation in vivo [36]. Previous studies showed that pollen of mitochondrial ATP synthase subunit has an important role in the development of the male gametophyte. FoF1-ATP synthase function disorders affect mitochondrial energy output, leading to anther dysplasia [37,38]. In this study, FoF1-ATP synthase showed upregulated in pollen of triploid poplar ‘ZH1’. It is suggested that the low fertility of ‘ZH1’ may be related to the high expression level of FoF1-ATP synthase. 

### 3.2. Allergenic Proteins Were Differentially Expressed in Mature Pollen of ‘ZH1’ and ‘ZH2’

Based on our previously identified 28 allergenic proteins in *P. deltoides* CL. ‘2KEN8’ [22], the expression patterns of allergenic proteins in ‘ZH1’, ‘ZH2’, and ‘2KEN8’ were compared. The expression of candidate allergenic proteins in ‘ZH1’ and ‘ZH2’ were lower than that in ‘2KEN8’. To a certain degree, it could be considered that the potential allergenicity of pollen in triploid poplar ‘ZH1’ and diploid poplar ‘ZH2’ may be lower than ‘2KEN8’. Compared with the diploid poplar ‘ZH2’, most allergenic proteins showed low expression in pollen of triploid poplar ‘ZH1’. It implies that not only germination, but also potential allergenicity of the pollen, in triploid poplar ‘ZH1’ were lower than diploid poplar ‘ZH2’. Cry j1 and Cry j2 are the major allergens in pollen of Japanese cedar (*Cryptomeria japonica*). Kondo et al. observed that the amounts of Cry j1 and Cry j2 proteins in the pollen of triploid Japanese cedar were less than diploid [39]. It could be deduced that the pollen in triploid might be less allergenic.

Based on the genome-wide prediction, Chen et al. predicted 145 and 107 pollen allergens from rice and *Arabidopsis*, respectively. These allergens are putatively involved in stress responses and metabolic processes such as cell wall metabolism during pollen development [40]. In the 28 candidate allergenic proteins from poplar mature pollen, several members belong to heat shock protein or profilin families. The class I small heat shock proteins (Hsps) were reported as one class of allergens in soybean [41]. Profilins are pan-allergen proteins present in various edible plant parts and pollen, such as birch pollen Bet v2, olive pollen Ole e2, grass pollen Phl p12, and soybean allergen Gly m3 [42,43]. Enolase is a glycolytic enzyme which was identified as a class of highly conserved fungal allergens, such as *Cladosporium herbarum*, *Alternaria alternata*, *Curvularia lunata*, *Penicillium citrinum*, and *Aspergillus fumigatus* [44,45,46]. Triosephosphate isomerase was described as allergen in wheat, latex, and lychee [47,48]. The remaining allergenic proteins are mainly involved in protein synthesis and degradation, but whether they can cause allergic reactions needs to be further studied. In this study, the expression of two Ole e1 were downregulated in pollen of triploid poplar ‘ZH1’ compared with the diploid poplar ‘ZH2’ (Table 2). Ole e1 is a major allergen which was first identified in olive tree pollen [49]. The proteins encoded by tomato *LAT52* gene and maize *Zmc13* gene also showed high similarity to Ole e1 [50,51]. Although Ole e1 plays important role in pollen physiology (e.g., hydration, pollen tube germination or growth, and other reproductive processes), its biological function is not yet known [52]. 

## 4. Materials and Methods

### 4.1. Plant Materials and Pollen Collection

Experimental materials in this study were collected from Chinese Academy of Forestry (Beijing, China). The triploid poplar ‘ZH1’ and diploid poplar ‘ZH2’ were obtained from the same parents by artificial cross-breeding. The cut flowering branches were cultured in water in greenhouse. At anthesis, fresh pollen was collected in the morning by shaking the tassel in a plastic bag, while old pollen and anthers were removed from tassels by vigorously shaking the evening of the day before.

### 4.2. Determination of the Ploidy Level

Root tips (0.5–1.0 cm) were collected from young plants and pretreated with saturated aqueous solution of *p*-dichlorobenzene (Sigma-Aldrich, Steinheim, Germany) for 3 h (room temperature), then fixed in Carnoy (glacial acetic acid:absolute ethanol 1:3) (Sigma-Aldrich, Steinheim, Germany) for 1 h. After treatments, the root tips were washed in distilled water, hydrolyzed in 1 mmol/l HCl (Sigma-Aldrich, Steinheim, Germany) for 45 min at 45 °C, stained using phenol-fuchsin solution (Sigma-Aldrich, Steinheim, Germany) and squashed [53,54]. Karyotype symmetry was classified according to Stebbins [55]. 

### 4.3. Pollen Germination

Pollen was germinated on liquid germination medium (15% sucrose, 100 mg/L H_3_BO_3_, 300 mg/L CaCl_2_, 200 mg/L MgSO_4_, 100 mg/L KNO_3_, pH 6.0) (Sigma-Aldrich, Steinheim, Germany) at 22 °C in the dark. Pollen of ‘ZH1’ and ‘ZH2’ was germinated in the same conditions. Pollen germination rates and the lengths of pollen tubes were measured microscopically after 2, 4, 8, 12, 24, and 48 h of incubation. For germination rates, each sample was observed in 15 fields of view. At least 30 pollen grains were analyzed in each field. Pollen grains were considered to be germinated when the pollen tube grows longer than the diameter of the pollen grain. For pollen tube growth, 50 pollen tubes were measured using ImageJ software (National Institutes of Health, Bethesda, MD, USA) [56] at each time point. All the experiments were performed in three biological replicates.

### 4.4. Protein Extraction and 2-DE Gel Electrophoresis

Protein extraction and 2-DE gel electrophoresis were performed as previously described [15]. Briefly, the total protein of pollen was extracted according to trichloroacetic acid (TCA)-acetone precipitation method, and the protein concentration was determined using the Bradford method [57]. The 2-DE was performed using the IPGphor system and IPG dry strips (18 cm, pH 4–7, nonlinear gradient) (GE Healthcare, Buckinghamshire, UK). For each sample, 450 μg of protein was uploaded on a strip which was saturated in rehydration solution. After isoelectric focusing (IEF), strips were immediately equilibrated for 15 min in a buffer containing 0.1 M Tris-HCl (pH 8.8), 2% (*w*/*v*) SDS, 6 M urea, 30% (*v*/*v*) glycerol and 0.1 M DTT, and another 15 min in the same buffer containing 0.25 M iodoacetamide without DTT. The second dimension was performed using 12.5% SDS-PAGE at 20 mA/bloc until the dye front reached the end of the gel in a PROTEAN II xi multi-cell (Bio-Rad, Richmond, CA, USA). After electrophoresis, the gel was fixed overnight in the stationary solution (50% (*v*/*v*) ethanol with 10% (*v*/*v*) orthophosphoric acid). And then transferred the gel to the mixed dyeing solution in the shock stained for 13 h and washed with water. Each 2-DE was repeated at least 3 times to ensure the reliability of results. The 2-DE images were assembled in a matchset using the Imagemaster 2D platinum 7.0 software (GE Healthcare, Little Chalfont, UK). After automated spot detection, the matched spots were verified and adjusted manually.

### 4.5. MALDI-TOF/TOF MS/MS and Database Search

Images of the stained gels were captured with a scanner (UMAX Powerlook 2100 XL; UMAX, Taiwan, China). Spot detection, matching, and background subtraction were performed using the ImageMaster 2D Platinum software (version 6.0; Amersham Biosciences, Uppsala, Sweden), followed by manual editing. All the spots detected in each gel were matched with the corresponding spots from the reference gels. To exclude the likely differences introduced by sample loading or gel staining/destaining, the normalized relative percent volume values (% volume) of the protein spots from three replicates were used for further statistical analysis. Selected spots were digested with gold grade trypsin, and then analyzed by a MALDI-TOF/TOF tandem mass spectrometer ABI4800 proteomics analyzer (Applied Biosystems, Framingham, MN, USA). For protein identification, the acquired MS/MS data were uploaded on the Protein Pilot software (Applied Biosystems, Framingham, MN, USA) and compared against *P. trichocarpa* genome (V3.0) database (https://phytozome.jgi.doe.gov/pz/portal.html). Proteins identified with a Mowse score ≥60 (*p* < 0.05) were reported. To annotate the identified proteins, the Gene Ontology (GO) was used to classify the proteins into three main classes, biological process (BP), molecular function (MF), and cellular component (CC). In addition, the enriched GO terms were slimmed in REVIGO web server [23].

### 4.6. RNA Isolation and qRT-PCR

Total RNA was extracted using the RNeasy Plant Mini Kit (Qiagen, Hilden, Germany) with on-column treatment using RNase-free DNase I (Qiagen, Hilden, Germany) to remove genomic DNA contamination. First-strand cDNA was synthesized with approximately 1 μg RNA using the SuperScript III reverse transcription kit (Invitrogen, Carlsbad, CA, USA) and random primers according to the manufacturer’s instructions. Primers for 28 predicted allergenic genes were according to Zhang et al. [22]. All the primer sequences used in this study are listed in Table A1. qRT-PCR was conducted on LightCycler 480 Detection System (Roche, Penzberg, Germany) using SYBR Premix Taq Kit (TaKaRa, Dalian, China) according to the manufacturer’s procedure. The *PtActin* gene was used as reference gene.

## 5. Conclusions

In this study, we analyzed the fertility and proteome of pollen in triploid poplar ‘ZH1’ and diploid poplar ‘ZH2’. Compared with the ‘ZH2’, the mature pollen in triploid poplar ‘ZH1’ failed to germinate. Through comparative proteomics, 67 of 72 DEPs were identified using MALDI-TOF/TOF MS/MS. The main functions of DEPs between triploid and diploid poplar pollen were “S-adenosylmethionine metabolism”, “actin cytoskeleton organization”, and “translational elongation”. Furthermore, the abundances of 28 putative allergenic proteins in three varieties (‘ZH1’, ‘ZH2’, and ‘2KEN8’) were compared. In short, not only fertility but also potential allergenicity of pollen were decreased in triploid poplar ‘ZH1’. This study is helpful for understanding the molecular mechanism of differences in pollen fertility and allergenicity between triploid and diploid poplar.

## Figures and Tables

**Table 1:** Differentially expressed protein spots (DEPs) identified in the pollen of triploid ‘ZH1’ and diploid ‘ZH2’ *P. deltoides*.

Spot Number	Gene ID	Annotation	Score	Coverage (%)	Experimental *M*_W_ (kDa)/Isoelectric Point (pI)	Theoretial *M*_W_ (kDa)/pI	Average Fold Change (‘ZH1’/’ZH2’)	*p*-Value
**Carbohydrate Metabolism and Energy Metabolism Related Proteins**								
29	Potri.010G027800	Pyruvate orthophosphate dikinase	663	57	28/6.07	39.78/5.64	−5.83	0.017
2	Potri.002G082100	PDI-like 1-2	396	41	50/4.58	43.88/4.64	−5.57	0.050
28	Potri.019G063600	pfkB-like carbohydrate kinase family protein	115	56	25/5.78	35.27/5.81	−5.04	0.049
15	Potri.015G131100	Enolase	184	71	42/5.88	47.74/5.66	−4.03	0.045
18	Potri.015G131100	Enolase	290	54	44/5.56	47.74/5.66	−4.03	0.046
41	Potri.002G181300	Pyrophosphorylase 1	400	46	23/5.98	24.88/5.92	−3.83	0.008
10	Potri.015G131100	Enolase	290	68	45/5.87	47.74/5.66	−3.64	0.041
49	Potri.002G134600	P-loop containing nucleoside triphosphate hydrolases superfamily protein	191	66	15/5.48	23.04/5.56	−3.40	0.012
43	Potri.006G082500	Pyrophosphorylase 4	322	48	20/6.66	24.92/5.92	−3.20	0.028
12	Potri.015G131100	Enolase	290	68	45/5.91	47.74/5.66	−2.91	0.044
31	Potri.010G117900	Aldolase superfamily protein	61	27	25/5.94	42.8/6.44	−2.83	0.030
27	Potri.001G061400	Transketolase family protein	396	47	24/5.53	38.85/5.87	−2.82	0.028
30	Potri.008G166800	Lactate/malate dehydrogenase family protein	206	49	29/6.55	36.3/6.61	−2.56	0.013
55	Potri.002G134600	P-loop containing nucleoside triphosphate hydrolases superfamily protein	63	41	12/5.57	12.18/5.45	−2.44	0.018
13	Potri.006G116800	Enolase	90	55	39/5.57	47.84/5.56	−2.17	0.041
14	Potri.006G116800	Enolase	175	60	39/5.85	47.84/5.56	−2.17	0.003
26	Potri.001G061400	Transketolase family protein	95	51	24/5.45	28.85/5.87	−2.05	0.016
52	Potri.003G060100	Rubredoxin-like superfamily protein	276	50	10/5.34	13.21/5.66	−2.03	0.015
11	Potri.017G144700	UDP-glucose pyrophosphorylase 2	118	47	34/6.44	51.74/6.78	−2.01	0.013
51	Potri.013G102100	Thioredoxin superfamily protein	173	46	11/4.97	11.92/5.32	−2.01	0.051
25	Potri.017G029000	pfkB-like carbohydrate kinase family protein	156	66	25/4.89	25.47/4.98	−2.00	0.030
50	Potri.001G173800	Rubredoxin-like superfamily protein	205	75	11/4.88	11.63/5.60	2.12	0.013
53	Potri.018G083500	Thioredoxin-dependent peroxidase 1	467	74	10/5.43	17.52/5.55	2.98	0.042
**Amino Acid Metabolism Related Proteins**								
22	Potri.002G189000	S-adenosylmethionine synthetase 2	72	47	35/5.49	43.61/5.62	−5.24	0.022
9	Potri.006G123200	Methionine adenosyltransferase 3	617	65	35/5.65	43.00/5.76	−4.69	0.018
19	Potri.006G123200	Methionine adenosyltransferase 3	617	65	35/5.65	43.00/5.76	−3.40	0.018
17	Potri.008G099300	S-adenosylmethionine synthetase family protein	483	79	33/5.48	43.60/5.50	−2.31	0.036
48	Potri.010G003500	Cytidine/deoxycytidylate deaminase family protein	90	59	16/5.18	20.74/5.26	−2.01	0.007
**Protein Metabolism Related Proteins**								
20	Potri.001G153000	Hyaluronan / mRNA binding family	65	28	35/6.21	39.79/6.25	−4.06	0.031
21	Potri.002G215900	GTP binding Elongation factor Tu family protein	70	43	35/5.78	49.29/7.66	−3.17	0.018
54	Potri.002G056200	Ribosomal protein L7Ae/L30e/S12e/Gadd45 family protein	311	65	10/5.44	15.66/5.48	−2.98	0.050
58	Potri.009G146200	60S acidic ribosomal protein family	127	30	11/4.33	11.39/4.36	−2.65	0.027
47	Potri.003G109200	Mitochondrion-localized small heat shock protein 23.6	118	52	16/5.13	23.96/5.36	−2.64	0.017
42	Potri.007G079500	Copper ion binding;cobalt ion binding;zinc ion binding	467	58	17/6.54	27.83/6.50	−2.56	0.015
7	Potri.003G006300	Chloroplast heat shock protein 70-2	593	39	75/5.14	75.41/5.24	−2.36	0.008
8	Potri.001G087500	Heat shock protein 70 (Hsp 70) family protein	142	39	75/5.18	84.74/5.04	−2.16	0.017
59	Potri.018G057600	Profilin 5	91	83	9/4.57	9.90/4.76	−2.14	0.011
44	Potri.018G063200	Chaperonin 20	227	68	18/6.34	26.87/6.64	−2.00	0.037
64	Potri.009G022300	Cystatin B	127	77	8/6.43	11.23/5.59	−2.00	0.026
67	Potri.009G039600	Tim10/DDP family zinc finger protein	109	66	6/5.45	9.86/5.56	2.00	0.026
36	Potri.011G089000	Co-chaperone GrpE family protein	79	48	18/5.12	34.58/6.04	2.01	0.010
37	Potri.015G122400	Proteasome subunit PAB1	147	54	17/5.23	21.73/5.95	2.01	0.012
38	Potri.015G122400	Proteasome subunit PAB1	147	63	17/6.09	21.73/5.95	2.02	0.012
57	Potri.004G187200	HSP20-like chaperones superfamily protein	121	36	11/5.34	13.05/5.54	2.06	0.035
40	Potri.006G008800	20S proteasome α subunit C1	404	74	20/5.87	27.55/5.96	2.08	0.038
62	Potri.012G039100	Tim10/DDP family zinc finger protein	161	40	8/5.79	11.23/5.81	2.10	0.001
23	Potri.009G018600	Glutathione S-transferase, C-terminal-like;Translation elongation factor EF1B/ribosomal protein S6	130	56	24/4.58	24.59/4.62	2.34	0.044
24	Potri.003G081000	Ubiquitin C-terminal hydrolase 3	313	43	24/4.65	21.64/4.78	2.71	0.036
60	Potri.003G047700	Profilin 3	411	58	9/4.72	14.21/4.71	4.10	0.021
61	Potri.001G190800	Profilin 1	132	51	8/5.26	14.22/4.75	5.59	0.013
**Defense or Stress Related Protein**								
16	Potri.003G113400	Stress-inducible protein, putative	106	39	61/6.22	65.81/6.17	-2.90	0.034
56	Potri.003G107100	Lipase/lipooxygenase, PLAT/LH2 family protein	139	48	8/5.83	10.37/6.05	2.00	0.015
5	Potri.007G024000	Late embryogenesis abundant (LEA) protein	162	44	34/4.61	44.97/4.64	2.12	0.016
6	Potri.007G024000	Late embryogenesis abundant (LEA) protein	721	57	34/4.75	44.41/4.81	2.30	0.021
3	Potri.007G024000	Late embryogenesis abundant (LEA) protein	124	47	48/4.98	44.41/4.81	2.34	0.034
63	Potri.013G031100	Copper/zinc superoxide dismutase 1	154	35	8/6.33	21.07/7.34	2.77	0.028
4	Potri.007G024000	Late embryogenesis abundant (LEA) protein	101	40	50/4.89	44.97/4.64	3.00	0.030
33	Potri.002G034400	NmrA-like negative transcriptional regulator family protein	280	60	25/5.62	23.96/5.51	3.11	0.338
66	Potri.004G107100	Late embryogenesis abundant protein (LEA) family protein	73	64	8/5.28	7.11/6.18	3.94	
65	Potri.017G108400	Late embryogenesis abundant protein (LEA) family protein	124	80	7/5.23	6.93/6.13	4.79	
**Signal Transduction Proteins**								
1	Potri.013G009500	Calreticulin 1b	86	39	47/4.02	51.46/4.92	−2.34	0.024
32	Potri.002G095600	Annexin 1	80	34	22/6.87	23.69/6.45	−2.10	0.019
**Allergy Related Proteins**								
45	Potri.001G392400	Pollen Ole e 1 allergen and extensin family protein	79	36	12/4.89	18.15/4.78	−5.47	0.018
46	Potri.011G111300	Pollen Ole e 1 allergen and extensin family protein	483	51	15/4.55	18.32/4.85	2.59	0.015
**Unknown Proteins**								
34	Potri.001G034400	Nascent polypeptide-associated complex (NAC), α subunit family protein	100	31	22/4.34	22.31/4.34	−3.84	0.038
35	Potri.004G135300	Catalytic LigB subunit of aromatic ring-opening dioxygenase family	171	69	23/4.87	29.67/5.88	−2.09	0.041
39	Potri.005G085500	Copper ion binding;cobalt ion binding;zinc ion binding	262	61	22/7.23	28.02/7.71	2.45	0.047

**Table 2:** Differential expression of 28 candidate allergenic proteins.

Spot Number	Gene ID	Annotation	Average Fold Changes		
			‘ZH1’/‘2KEN8’	‘ZH2’/‘2KEN8’	‘ZH1’/‘ZH2’
1	Potri.003G006300	Chloroplast heat shock protein 70-2	−1.48	−1.30	−2.59
2	Potri.001G087500	Heat shock protein 70 (Hsp 70) family protein	−1.88	2.87	−5.57
3	Potri.001G285500	Mitochondrial HSO70 2	1.49	−1.72	3.33
4	Potri.009G079700	Mitochondrial HSO70 2	−1.42	−1.16	−1.31
5	Potri.006G116800	Enolase	−4.08	−1.17	−2.17
6	Potri.015G131100	Enolase	−2.06	−3.89	−2.91
7	Potri.019G067200	Pectin lyase-like superfamily protein	–	–	–
8	Potri.002G034400	NmrA-like negative transcriptional regulator family protein	2.19	3.48	−1.29
9	Potri.012G114900	Pectin lyase-like superfamily protein	4.81	6.55	−3.10
10	Potri.010G117900	Aldolase superfamily protein	–	–	–
11	Potri.001G034400	Nascent polypeptide-associated complex (NAC), α subunit family protein	−3.56	−1.98	2.88
12	Potri.009G018600	Glutathione S-transferase, C-terminal-like;Translation elongation factor EF1B/ribosomal protein S6	2.17	4.57	−2.37
13	Potri.008G056300	Triosephosphate isomerase	−3.61	−1.74	3.12
14	Potri.013G092600	Manganese superoxide dismutase 1	1.03	−1.52	0.77
15	Potri.001G392400	Pollen Ole e 1 allergen and extensin family protein	3.86	4.07	−1.37
16	Potri.011G111300	Pollen Ole e 1 allergen and extensin family protein	1.37	1.54	−1.62
17	Potri.009G146200	60S acidic ribosomal protein family	–	–	–
18	Potri.006G235200	Profilin 4	-4.10	−3.45	−1.99
19	Potri.018G057600	Profilin 4	–	–	–
20	Potri.003G047700	Profilin 3	−6.93	−3.48	4.10
21	Potri.001G190800	Profilin 5	−3.14	−1.36	5.59
22	Potri.007G018000	Thioredoxin H-type 1	−2.29	−1.33	−3.19
23	Potri.018G083500	Thioredoxin-dependent peroxidase 1	−1.11	−3.55	3.97
24	Potri.009G147900	HSP20-like chaperones superfamily protein	−4.63	−6.33	−2.79
25	Potri.001G254700	HSP20-like chaperones superfamily protein	–	–	–
26	Potri.006G093500	HSP20-like chaperones superfamily protein	–	–	–
27	Potri.001G254700	HSP20-like chaperones superfamily protein	–	–	–
28	Potri.005G232700	Thioredoxin H-type 1	0.36	0.89	−0.63

## Appendix A

**Table A1:** The sequences of qRT-PCR primers.

Spot Number	Gene ID	Forward Primer	Reverse Primer
3	Potri.001G285500	TTTGGAAAGAGCCCTAGCAA	CCTGAGTCTGGTTGTCAGCA
10	Potri.010G117900	TTTTTGTCTGGAGGGCAATC	TTTCACCCTCGGCAGAATAC
15	Potri.001G392400	CCAAAATCAGCGAGGGAATA	AGAATCAATGCTCCGGAATG
16	Potri.011G111300	TGCTTCTCCTCCGTTCTCAT	GTCAATGTGCCATTCTCACG
21	Potri.001G190800	ACATGGTGATCCAGGGAGAG	GAGCACCAGCATTACCCTTC
28	Potri.005G232700	TCGAGAAGGGAAAAGGGTCT	ATTGCCTCCACATTCCACTC
Reference	Actin	GTGCTTCTAAGTTCCGAACAGTGC	GACTACCAAAGTGTCTGACCACCA
